# Evaluating interventions with victims of intimate partner violence: a community psychology approach

**DOI:** 10.1186/s12905-021-01268-7

**Published:** 2021-04-06

**Authors:** Cinzia Albanesi, Carlo Tomasetto, Veronica Guardabassi

**Affiliations:** Dipartimento di Psicologia, Università di Bologna, Viale Europa 115, 47521 Cesena, FC Italy

**Keywords:** Domestic violence, Intimate partner violence, Empowerment: IPV program

## Abstract

**Purpose:**

Intimate Partner Violence (IPV) is one of the most common forms of domestic violence, with profound implication for women's physical and psychological health. In this text we adopted the Empowerment Process Model (EPM) by Cattaneo and Goodman (Psychol Violence 5(1):84–94) to analyse interventions provided to victims of IPV by a Support Centre for Women (SCW) in Italy, and understand its contribution to women’s empowerment.

**Method:**

We conducted semi-structured interviews with ten women who had been enrolled in a program for IPV survivors at a SCW in the past three years. The interviews focused on the programs’ aims, actions undertaken to reach them, and the impact on the women’s lives, and were analysed using an interpretative phenomenological approach.

**Results:**

Results showed that the interventions provided by the SWC were adapted according to women's needs. In the early phases, women’s primary aim was ending violence, and the intervention by the SCW was deemed as helpful to the extent it provided psychological support, protection and safe housing. Women’s aims subsequently moved to self-actualisation and economic and personal independence which required professional training, internships, and social support. Although satisfying the majority of the women’s expectations, other important needs (e.g., economic support or legal services) were poorly addressed, and cooperation with other services (e.g., police or social services) was sometimes deemed as critical.

**Conclusions:**

By evaluating a program offered by a SCW to IPV survivors through the lens of the EPM model, we found that women deemed the program as effective when both individual resources and empowerment processes were promoted. Strengths, limitations and implications are discussed.

**Supplementary Information:**

The online version contains supplementary material available at 10.1186/s12905-021-01268-7.

## Background

According to the World Health Organization multi-country study on women’s health and domestic violence against women, the number of women who have experienced physical violence from a partner ranged between 4 and 49% across countries, while 20–75% reported experiencing one emotionally abusive act or more from a partner in their lifetime [15]. Intimate Partner Violence (IPV) is one of the most common forms of violence against women, including those who are the parents or primary caregivers to minor children. IPV has been shown to be more prevalent among couples with children compared to couples without both in the U.S. [23] and in Europe [12]. In Italy, 65% of IPV victims had children [16].

The consequences of IPV on women and children can be dramatic. Women experience increased risk of depression, anxiety, substance abuse, post-traumatic stress disorder [11, 32, 35], and reduced confidence in parenting skills (i.e., lack of emotional support for their children and responding adequately to their needs, [26]). Witnessing IPV has immediate and long-lasting detrimental effects also on children, including anxiety, depression, and psychosocial problems [6, 13, 14, 22].

The positive effects of programs to help IPV survivors have been documented. For example, Trabold et al. [39] showed the efficacy of a trauma-informed brief intervention on victims of IPV. The program is based on social-cognitive therapy, is composed of 10 sessions, and seeks to improve the condition of IPV victims in terms of physical health and psychological well-being. Similarly, 10 or 12 sessions of narrative exposure therapy (a treatment for trauma disorders conceived by Schauer et al. [31]) were found to have a positive impact on post-traumatic stress disorder symptoms, depression, and perceived stress [24]. Kokka et al. [19] showed different benefits of a stress reduction program for IPV victims (lower levels of depression and anxiety, increased self-esteem, self-efficacy, and health-related behaviours). More specifically, Sprague et al. [34] reviewed 43 studies (65% in North America, 16% in Europe, and 12% in Australia) presenting programs delivered within health care settings in different countries. Their analyses revealed that most programs were beneficial for women, even though they largely differed in terms of type of help provided (i.e., active vs passive assistance, single type vs multiple types), the level of intervention (only one vs multiple types), and the outcomes considered (re-victimisation vs well-being measures). Trabold et al. [38] showed indeed that programs to help survivors are very diverse in terms of theoretical frameworks and models of care. The most promising were cognitive programs focusing on psychopathological outcomes, well-being therapy, interventions focused on resource mobilisation, social support, problem-solving focused on re-victimisation, and advocacy and empowerment programs. Whilst different programs showed promising results worldwide, their strengths and weaknesses are not always clearly understood. This is the case in particular for the programs offered in Italy by Support Centres for Women (SCW) (in Italian, *Centri Anti-Violenza*, litt. Anti-Violence Centres) that are based on the principle of women’s empowerment [27].

### Empowerment programs for victims of IPV

Empowerment is a common framework for programs aimed at IPV survivors, in particular in Italy. However, Kasturirangan [18] outlined that in many cases IPV programs do not provide a clear definition of empowerment, equating the term with “advocacy” or “enlarging the basket of opportunities” for women. Instead, “program goals should be shaped by women’s own values and priorities and should reflect the limitations to resource access placed on them by society. Programs must acknowledge that a woman who places her family’s well-being before her own, or rejects options laid in front of her because they will not work for her circumstances, may still be engaging in an empowerment process. Women who engage in an empowerment process should set their own goals and determine what kinds of resources would be helpful to them in reaching these goals” [18] p. 1469]. Clear conceptualisation is indeed critical to support an authentic empowerment process and evaluate its results. 

Cattaneo and Goodman [9] p. 84] defined empowerment as “a meaningful shift in the experience of power attained through interaction in the social world.” This broad definition, based on the Empowerment Process Model (EPM, Cattaneo and Chapman [8]), assumes empowerment as an iterative process where internal (psychological) and external (social) factors interact, and in which “a person who lacks power sets a personally meaningful goal-oriented towards increasing power, takes action and makes progress towards that goal, drawing on his or her evolving self-efficacy, knowledge, skills, and community resources and supports, and observes the impact of his or her action” [9], p. 88].

Building upon consolidated literature on empowerment (e.g., [28, 40]), the EPM includes three focal components, namely:

- *Meaningful, power-oriented goals and objectives.* The identification of personal goals through the exploration of possibilities and the selection of the best option—based on the unique circumstances each survivor faces at any given time—is essential to the process of empowerment. This component often also involves revisiting goals and sub-goals over time, as the context and other components of the process shift.
- *Actions toward goal achievement.* The second component refers to all the activities that are necessary to reach the selected aims. Individuals need to trust their *knowledge*, i.e., their understanding of what must be done in order to reach goals. It is distinct from the ability to actually take those steps, which is determined by *skills*, as well as by the perception of being able to do it (i.e., *self-efficacy*). The possibility of reaching personal aims also depends on building and/or accessing community resources. Formal systems, as well as informal support (e.g., friends, neighbours, co-workers, or family), are also part of the process of empowerment.
- *Observation and reflection on the impact of actions in relations to established goals*. The last component concerns the individual’s awareness of one’s progress in terms of internal experience and external changes. The impact is context-specific, as it is deeply related to individuals' goals.

Contextual variables influence all the components of the process: cultural values can modulate meaningful personal goals, and some contexts more than others can facilitate access to psychological and social resources. Thus, the possibility of carrying out certain actions or attaining certain aims also depends on the social context in which individuals live.

In order to consolidate the theoretical foundation of empowerment, Brodsky and Cattaneo [5] distinguished its convergence and divergence with respect to resilience. Resilience and empowerment are two interactive processes which both build on personal and social resources (e.g., knowledge, skills, self-esteem, and social support). However, they occur under different circumstances that define the magnitude and the transformational capacity of (social) change. For example, resilience occurs when the level of risk is high, the magnitude of change is large (e.g., become a feminist activist in a non-democratic country where women have limited rights), and the only possible change can be internal or local (e.g., adaptability or resistance) [5]. When the level of risk is low (e.g., supportive laws exist), the magnitude of change is smaller (e.g., become a feminist activist in a democratic country with gender disparity), and transformative actions are possible (e.g., claiming rights), it is the empowerment process that takes place [5].

Cattaneo and Goodman [9] developed a community psychology framework based on EPM to guide intervention and to assess programs’ effectiveness in empowering women victims of IPV (see Table 1).

**Table 1:** Recommendations for applying the empowerment process model to Practice in IPV

Model component	Questions to guide evaluation
Goals	• What is the fit between survivor-defined and system-defined goals and subgoals? • To what extent do evaluation plans address survivor-defined versus system-defined goals and subgoals? • **What kind of formal and informal supports facilitate progress toward which kinds of goals and subgoals?** • **How do individual and contextual factors shape goal formation and goal achievement?**
Knowledge, skills, self-efficacy, and community resources	• **To what extent do programs facilitate attainment of resources in each of these areas?** • To what extent do specific strategies work to enhance these components? • What is the relationship between changes in one component and changes in another? • **How does trauma history influence the process of change in each of these areas?** • **What obstacles do survivors commonly face in growing or accessing resources in each of these areas?** • How does culture and other contextual factors influence change in these areas?
Impact	• **Beyond system-defined outcomes, how do various services affect outcomes most important to survivors?** • What kinds of outcome measures are most relevant to survivors’ goals, account for unintended negative consequences, and incorporate elements of the entire empowerment process, including both psychological and social components?

*Note* Questions in bold were selected to guide the evaluation of the SCW program in the present study

Specifically, the EPM framework identifies questions that can be used to explore goal identification, as goal setting is a cornerstone for women's empowerment and, consequently, for programs addressing IPV. Other questions aim to understand how/whether programs facilitate access to community resources and the development of psychological ones (knowledge, skills, self-efficacy). Resources are crucial for understanding how women can plan their actions and achieve their meaningful goals. Finally, specific questions allow evaluating the impact, i.e., the capacity of the program to support a reflexive process on one's progress, both in terms of internal experience and external change and on other unexpected (positive or negative) consequences.

In conclusion, the community psychology framework based on EPM appears to be a promising tool for evaluating the empowerment capacity of IPV programs and supporting their improvement.

### The present study

In this paper we used the Cattaneo and Goodman [9] EPM framework to analyse the strengths and weaknesses of the program for IPV victims at a Support Centre for Women (SCW) located in the Centre-North of Italy, and its contribution to women’s empowerment. The SCW, like most Anti-Violence Centres in Italy, is run based on the principle of women’s empowerment [27]. The Centre asked the Department of Psychology of the University of Bologna for a formative evaluation of the program offered to women victims of IPV [30]. The SCW was interested in improving how it operates/functions, removing obstacles to access, and identifying good practices based on successful stories of women who survived IPV. Given the emphasis on empowerment as a driving principle of SCW interventions, we identified the evaluative framework developed by Cattaneo and Goodman [9] as particularly useful to shed light on the strengths and weaknesses of the SCW program. We selected a set of questions from the EPM framework that we identified as the most suitable for a qualitative formative evaluation based on the users’ perspective. These questions were then used in order to evaluate the capacity of the program to help women reach their desired goals, to mobilise resources, and to support women's reflection on their personal trajectories and their life context. Formative evaluation focuses on how the program or service is experienced by participants [25]. This may be especially important with regard to services for women victims of IPV, as there is evidence that how services are delivered is as important as the kind of services that are offered [1]. This type of evaluation generates data that can immediately benefit the service and its potential users [17]. The selected questions are in bold in Table 1.

## Method

### Participants and procedure

Participants eligible to take part in the study were women survivors of IPV who were mothers, who had turned to the SCW for help between 2015 and 2017, and who were still in contact with the Centre. The SCW contacted eligible participants by telephone to verify their willingness to be interviewed by the research team about their personal story of violence. Fifteen women volunteered to participate and gave their consent to be contacted by the research team. We then proceeded to contact them by phone to explain the aim of our project and the procedure of the study and, if the women agreed to participate, we arranged an appointment in order to conduct our interview at the Support Centre. In this phase four women decided not to take part in the research due to work or family issues, and one woman could not be contacted by phone. Thus, our final sample was composed of 10 women survivors of IPV. Neither monetary nor any other form of compensation was provided. Participants, however, were informed of the practical implications of the study and the importance for researchers to understand the IPV victims’ perspective.

### Data collection

The interviews took place at the SCW on pre-arranged dates in June and in July 2018. Participants received an oral and written explanation of the aims of the study and the content of the interview, and provided written informed consent for their participation. The research followed the code of ethics of the Italian Association of Psychology [3], and received approval from the bio-ethics committee of the University of Bologna. Data were collected using a semi-structured interview. The interview guide was purposely developed for this study. Each interview was conducted in Italian by two members of the research team, and took an average of about one hour. Specifically, V.G., who holds a PhD in Psychology, conducted the interviews with the support of a junior member of the research team with a Bachelor's degree in Psychology. The junior member of the team was in charge of taking field notes, observing non-verbal interaction, and of the verbatim transcription of audio-recordings. All the interviewers were female and white. The junior members of the research team took part in the research for their final dissertation, in partial fulfilment of the requirements for their Master's degree in Psychology. All the team members were motivated by an interest in the research and had a robust background in gender studies.

Questions about socio-demographic background, hobbies, and personal interests opened the interview to break the ice, before turning the conversation to more personal matters. The interviewer introduced herself when opening the conversation, and further clarified the aims of the study. The next set of questions was about their experience at the SCW and with other institutions (e.g., social services, health services, Police, courts etc.), specifically focusing on what occurred before, during, and after the conclusion of the program (e.g., changes, impact, what worked and what did not). The interview also focused on their experience as mothers, as victims of a violent partner, and as women who survived IPV and moved on. The interviews were audio-recorded and transcribed verbatim, reviewed, and checked for errors. They were not returned to participants for comment, but they were complemented with field notes and observations from the junior member of the research team.

### Data analysis

For our analyses, we adopted an interpretative phenomenological analysis (IPA) [33] framework, which is considered an appropriate approach to investigate individuals’ experiences related to a particular phenomenon, and the meaning that participants attribute to their experiences [20]. First, we proceeded with several readings of the transcripts and we coded all the content in detail using a text editor. Coding was initially almost unstructured, with short notes added to identify core points in women’s stories (i.e., the “turning points” of their experiences), capture their emotions, etc. Then we progressively organised the initial coding into themes. Specifically, we first highlighted those parts of the texts from each interview, which we found relevant for a specific theme, then we collected all the relevant quotes that belonged to the same theme from each interview. Finally, the themes were refined through a process of reiterated comparisons across the interviews and between the researchers, in order to identify overarching themes that could “dialogue” with the theoretical models. A spreadsheet was also used. For example, it became clear from the very preliminary analysis that support was a core dimension of women’s experience and a fundamental resource. Support was coded at the beginning according to the specific source (e.g., support from family, support from friends, support from the SCW), and then into broader categories (e.g., informal and formal support; satisfactory and unsatisfactory). The categories were then further analysed to cluster and establish relationships among them, in order to provide answers to the main questions that drove the evaluation according to the EPM model: basically, detecting the women’s meaningful (i.e., power-oriented) goals, how they were achieved, the role played in this process by different resources (knowledge, skills, self-efficacy and community resources), and the impact this had on their lives.

The analysis was conducted by three members of the research team who started working on the analysis independently. To ensure rigour each research team member coded the interviews independently. Coding was then discussed and an agreement reached under the supervision of two senior researchers (C.A., C.T.). The senior researchers (one woman, one man) were both white, married, the parents of minor children, and hold a permanent position at the University. They played the role of “critical friend” in the research group, by posing provocative questions in order to engage junior researchers in a reflexive process of analysis that took into account their positive and negative emotions and thoughts.

This process, albeit time-consuming, allowed researchers to explicit their premises and the assumptions that drove interpretations, thus allowing them to solve (or account for) discrepancies through discussion, and identify recurrence across interviews with regard to categories and themes. Data saturation was reached when further coding/new coding categories were not needed/added after seven interviews had been analysed.

The results of the analysis were discussed with the professionals of the Support Centre, and with a couple of participants before they were finalised. For the purposes of this paper quotes in Italian were translated into English by the research team.

## Results

### Participants’ description

Data regarding the women's age, home country, educational level, employment, number of children, kind of violence experienced and medical reports were provided by the SCW with the participants’ signed consent. Participants’ age ranged between 26 and 46 years (*M* = 37 years, *SD* = 7.08). Five women were of Italian origin, three came from other European countries and two from African countries. Level of education ranged from low to medium. One woman had completed elementary school, three middle school, five high school, and one had a university degree. Two women had one child, seven had two children, and one had three children. All of them were victims of psychological and physical violence (four out of 10 had medical reports as a result of the violence against them), but there were also other forms of violence: Two women were victims of sexual violence, seven of financial abuse, and two were victims of stalking. At the time of their first access to the SCW, the majority of the women (eight out of 10) were unemployed. At the time of the interviews eight women out of 10 had stable employment in low-skilled positions, except for one who was employed in a bank.

### Thematic analysis

### Goals

The first theme of Cattaneo and Goodman’s [9] model included the goals and sub-goals of women victims of IPV, and how these goals changed through their involvement in the program. Specifically, most women indicated that one of the main goals at the time of their first contact with the SCW was to change their condition: Not in terms of improving their lives, but merely to stop their partner’s violence. In fact, they usually turned to the Centre after an extremely violent episode which made them more aware of their condition, or simply because they realised that they could no longer stand this reality. Eight women out of 10 asked for support from the SCW after more than five years of violence. Only one asked for support within one year, and one within five years.

Participant G: “There weren’t only arguments, but he was depressed, so it was a thing that I wasn’t able to manage, then he became violent and he broke my arm. It was because of this that I came here [SCW], because I could no longer.…”

Participant B: “That was not a life, I used to be on the alert, I used to be scared, I didn’t know where to go, but better out than in that house with that man.”

Thus, the most important goals in this phase were to be welcomed and protected (*acceptance* and *protection*), as well as having someone on one’s side (*psychological support*) to provide advice on what to do (*information*).

Participant L: “Yes, yes. I felt welcomed, they gave me much hope”

Several participants acknowledged that their needs evolved over time, and other goals progressively became more relevant, including the idea of improving their condition (*self-actualisation*), i.e., increasing their skills, obtaining a job in order to fend for themselves (*economic independence*), as well as maturing, being able to establish positive relationships, being good mothers, and increasing self-esteem and self-efficacy (*psychological well-being*). In some cases, when realising that they could not obtain everything at the same time, women prioritised specific goals:

Participant I: “No, I haven’t stopped taking care of myself. The only thing that I think of, at the moment, is to find a proper job and try to give, as much as possible, what I can to my children. Then, everything else doesn’t matter. It is logical that aging alone is scary, but I am not 20 years old, I am already 42.”

Participant B: “No, I never had any further contact [with the SCW] because I have several obligations now, work, my daughter’s skating course, there are some obligations.”

Participant E: “It is a good starting point [asking for initial help from the SCW], but, of course, you have to improvise a little bit to become someone else, and in any case you must become someone else, because either you have broad shoulders, or they will become broad anyway.”

What kind of formal and informal support facilitates progress towards which kind of goals and sub-goals? Formal and informal support played a role in goal achievement.

#### Formal support

Formal support was provided by Health Services (i.e., hospitals and clinics), the SCW (i.e., the Centre and safe shelters), and Social Services (i.e., social workers dealing with social, psychological and economic difficulties). All these services are accessible for free, and their functioning is guaranteed by local (e.g., Region, Province, or Municipality) or national (e.g., Ministry) institutions. Formal support was provided in different ways. SCW and other Services offered welcome and refuge to women who had moved away from their violent domestic contexts. Four women went directly to the SCW while others asked other services for support (i.e., Child Protection Services, Police, hospital) and were subsequently directed to the SCW. The Centre offered initial help by welcoming the women. Individual counselling or self-help group opportunities (i.e., psychological support) were then offered, which were important to sustain women’s self-esteem and self-efficacy, as well as to reduce their sense of loneliness *(self-actualisation, psychological well-being)*.

Participant H: “For example, meeting with other mothers helps you, because there are many stories, each one different from one another, but they all have something in common. For example, being a single mother or to have had problems with violence. So, you realise that you aren’t the only one, there’re lots of girls in the same situation, and this helps you, also to talk and be confident that we can change.”

In addition, the SCW also gave them the opportunity to consult a lawyer with regard to their legal status *(information),* and helped to arrange training courses or internships to facilitate their entry into the labour market (*self-actualisation; economic independence*). In cooperation with other local resources, women were also directed to different services based on their specific needs (*information*). For example, the health services provided medical treatment (e.g., treatment for injuries or assistance during pregnancy), while social services offered tutoring for children or housing support, economic help (*social support*), as well as housing solutions to protect them from the perpetrator of violence (*protection*). Similarly, the SCW worked with the law enforcement authorities to provide *protection* by removing the violent partner or housing the women in protected shelters.

Participants declared they had felt *accepted*, *psychologically supported* and that they had received useful *information* from professionals (with only two negative reports regarding social services). However, participants also underlined some limitations. First, some women criticised the effectiveness of the legal service because the list of available lawyers was not updated. Second, bureaucracy lengthened the time needed to have paperwork completed, so that the achievement of women’s goals was more complex than expected (e.g., obtaining a house). Third, attending an internship was complex for women lacking help for children care, and required a lot of time without any assurance of future employment. Finally, other participants underlined the limited economic support, which was deemed insufficient to fulfill housing needs for them and their children.

Participant E: “They [the SCW] show you a list of lawyers, but when you speak with one of them, they aren’t in the list anymore or maybe they dissuade you, or they don't work anymore in these cases because the State [could no longer deal with it]. So, it's always because of bureaucratic issues. In the end, you'll become discouraged, and you'll decide to access to a lawyer on your own.”

Participant G: “They [children] want to invite friends at home, but where is the home? It’s a hole where we try to survive. We are not living; we are surviving here.”

Nevertheless, the help received from the SCW was evaluated positively by the majority of the participants (eight out 10) who defined the Centre as a point of reference with very competent professionals who do their best.

Participant C: “At the Centre, yes, very, very good! I was really a mess! I had trouble working. It was useful and beautiful too! The girls here [professionals at the SCW] are very kind and helpful.”

Participant D: “Yes, they [the SCW] put effort into looking for your economic and residential independence. However, this is very slow because maybe they aren’t supported, they cannot do more, and we’ll see what happens!”

Conversely, not all the opinions concerning the *sense of protection* were positive. On the one hand, safe housing was viewed as useful by some women as they could have their own place as well as living away from the perpetrators of violence.

Participant A: “I don’t know, I couldn’t sleep when I was with my husband, I put my head [on the pillow], but I couldn't sleep, as I was worried that he could arrive and hurt me with something, because he tried to do this. But, when I was here [in a house provided by the SCW], I was able to sleep. During the day I was a little bit sad, but during the night I was able to sleep.”

On the other hand, women sometimes reported their fear of being chased by their partner, because even though they did not know where they were living, they knew where they worked or where their children usually went.

Participant E: “Yes. No. When you walk, you are afraid as well, you are anxious, you are afraid when you see some cars, but fortunately, now, I got rid of this thing. However, I believe that it’s hard to feel safe at first, it always seems that someone is behind you.”

In line with these considerations, some women also explicitly referred to the work that law enforcement does with the SCW. Only one participant gave a completely positive evaluation, while two offered a mixed evaluation, and another two women expressed an overtly negative view.

Participant E: “There was significant collaboration with the central Police station. It was a smooth collaboration, that allowed me to leave my home in an appropriate way.”

Participant D: “Once the restraining order was issued, they stopped the investigations and that circle [of collaboration between the SCW and Police] was interrupted. A link of the chain has broken and consequently, that sense of protection wasn't there anymore.”

Participant I: “That billboard which suggests women to report [violence to the Police] should be taken down because until a woman's face is battered and bruised, she is not believed. Don't go to the Police or to the central Police station, because you will obtain nothing.”

#### Informal support

Informal support was given by family, friends or colleagues. Four women declared that no one gave them any support. For example, one woman reported that her community had negatively evaluated the separation from her husband, and another one declared that friends had disappeared when they were asked to testify. To the contrary, other participants acknowledged that they had felt the closeness of their network of relationships: they were advised to go to the SCW (*information*), received hospitality, loans, favours (e.g., payment of a skating course for a woman’s child) or, simply, support for their decisions. Informal support facilitated "normalisation", providing opportunities for intimacy and positive experiences, especially for the children, and momentarily buffering economic and relational difficulties, or being helpful in reaching the goal of *self-actualisation.*

Participant C: "Yes, no, no, they [my relatives] knew that I was coming here, that I felt better. So, they were happy.”

Participant E: “I came here [to the SCW] thanks to the support of my sister and of one friend who is now my partner. Just then, he was only a friend. He helped me a little bit during the program; he supported me.”

Special reference also needs to be made to the presence of children. Some women pointed at their children as the main reason for changing their condition. On the contrary, others declared that it would have been easier to separate from their violent partner if they had not been mothers.

Participant F: "I didn’t react because I was afraid that after my legal action he could take him [the son] away, because he [author of violence] worked and I didn’t, I don't have relatives, I didn’t know where to go, so my mind said that he would get the custody of the child. Then, I closed my eyes, and I said: "I don't know what will happen, but in any case, I’m moving on!”

Participant L: "However, when it happens [violence] and when it doesn't work [the relationship with the partner], having children and not having anyone to turn to, it isn't easy. But I could no longer watch the children suffering.”

From the analysis of the interviews a change in the women’s idea of their responsibility as mothers emerged. At first, when the women were suffering violence, they held family cohesion and financial security as a priority. Offering a good parental model to their children and a stable context in which to grow up was seen as the most rational choice: This implied accepting the violence, choosing to conceal some events from their children, and offering their partner a second chance again and again. Later on they realised that family and couple relationships require reciprocal respect and responsibility and sharing common goals (i.e., taking care of children). Once this awareness emerged, these fundamental principles overcame other considerations, such as family cohesion and financial security. When they realised that their relationship would not change, that living in a violent environment was not positive for the children, and that the children would not be respected and protected by their father (e.g., with children witnessing violence or being themselves abused), they decided to make a change, at the cost of “moving” toward uncertainty.

#### How do individual and contextual factors shape goal formation and goal achievement?

Several women declared that they accessed the SCW in a very confused state, without any expectations, and with no idea of what would happen. This situation did not allow the professionals to set specific goals at the beginning of the case management process, but required them to set goals gradually together with their clients, while women progressively recognised their needs and resources. Thus, goal formation was the result of a mutual process where professional help was based on the women’s growing awareness of their condition, and their increased trust in the SCW. For this reason, each goal was defined a step at a time, based both on the women’s and the professionals’ perspectives.

Participant D: “Like a baby who takes his first steps, what does he do? He holds onto one side, he tries to reach the other side, he holds on and he makes his first steps. You feel this way, you feel that you have to leave something, but you need to lean on something else, not to walk into a vacuum.”

If a woman aimed at economic independence, professionals helped her increase her awareness of the realistic possibilities of getting job by analising her competences, the labour market etc. Based on this analysis, the following step might include enrolling the woman in either a professional training course or a paid internship, or directing her to an employment centre or helping her with a job interview. Even though each woman established her own individual goals, some goals were generally prioritised over others: protection (for the woman and her child) and safety usually came first, then independence and/or normalisation next, and finally self-actualisation may or may not be included as the final goal.

Goal achievement was based on individual as well as contextual factors. Specifically, *sense of protection* depends on the woman’s fear (individual factor) but is also tightly intertwined with the support received from the Police, violent behaviour of the ex-partner, and the availability of informal support (contextual factors). *Economic independence* is a complex goal that builds on competences, knowledge, and self-efficacy, which in turn can contribute to obtaining a job (individual) or doing an internship or a course (contextual). The process of *self-actualisation* builds on psychological support offered by the SCW, on legal support and on welfare facilities, but also heavily relies on the woman’s self-esteem and knowledge (individual).

Participant E: “I struggled at first taking small jobs here and there. Fortunately, I have always been a bit of an ant, a saver; so, I cashed in insurance policies, I sold my gold, and I succeeded [to economically support myself], also because I had my rent to pay, I had 500 euros to pay.”

Participant H: "Yes, of course. They give you useful support, especially if you give yourself a challenge.”

### Knowledge, skills, self-efficacy, and community resources

According to the EPM model, individual and community resources contribute positively to the empowerment process. Thus, we refer to *knowledge* as the acquisition of new information useful for achieving personal goals, including self-awareness; to *skills* as individual competences (e.g., professional competences, second languages, etc.); to *self-efficacy* as the perception of being able to do whatever is necessary in order to reach goals; and to *community resources* as formal and informal support.

*To what extent do programs facilitate attainment of resources? *Analyses showed that nine out 10 women gave the SCW full credit for teaching them something, for having made them more able to cope with everyday life, for being more aware of their own stories, and becoming aware of their strengths rather than only their limits (*knowledge*).

Participant A: “At first, when I was there, I’d almost given up hope, but, after that, I slowly settled down, thanks to the help they gave me that was useful, and that taught me how I could manage by myself.”

Participant I: "It was like I fell into a sinkhole and was unable to climb back out. Because several times I came here [to the SCW], I talked with [one professional], and she said something, but you hardly believe it, because you are totally submerged in the cauldron, and you have no awareness. Then, step by step.”

Women seem to attribute an important role to the SCW program in increasing their *self-efficacy* by becoming more aware of their personal story or, more generally, of themselves.

Participant E: “[The SCW] is a good starting point. Also, for the suggestions and everything. It’s a good starting point, but then you need, you know, to improvise and to be another person.”

The SCW, in cooperation with other services, also offered the possibility to develop some *skills* in professional and everyday life: A course in Italian language for one of the women who was interviewed, and internships for another two that evolved into two job positions.

Participant B: “However, that course was useful because I got a job that I still have, because I met good people.”

Finally, being involved in the programme gave the women the possibility to take advantage of the *community resources*. At first, for the majority of them, the SCW became a reference point they could rely on when they needed.

Participant D: “It’s the only point of reference at that moment. At least in my case, not having anybody [no relatives]. If you had something to say, you said it to them, and you talk about your situation. So, you do form a kind of relationship with professionals who follow you.”

Women had the opportunity to build relationships with other victims of IPV, whom they met in the psychological support group, or during training courses and internships.

Participant C: “It was a very difficult period, but being in a group, meeting other girls in similar situations, making friends was positive. We’re still in.”

In addition, for some women, the interruption of their relationship with the partner and the start of the program contributed to rebuilding their relationships with their family of origin or with old friends. Some of the participants could rely again on their informal support.

Participant F: “My friends, yes! They said “Oh, finally!” Everybody said “Oh, finally, you’re finally back!”

Participant A: “My brother-in-law and my mother-in-law, they were kind and they helped me, they were completely supportive and they didn’t get along with him [perpetrator of violence].”

*How does trauma history influence the process of change in each of these areas? *More than trauma history, it was the *awareness* of trauma history that influenced the process of change. Indeed, while the trauma slowed down the process of change, the awareness of trauma represented a turning point. Participants realised that they had been living in a condition of violence for a long time and, as they were not aware of the gravity of their situation, they had waited too long to ask for help.

Participant D: “But at that moment you cannot understand, you can’t manage. It’s this: You have to wake up from this thing, and, unfortunately, this takes time. You live with violence and you don’t say “hey, what are you doing?” You live a life, you adapt to it, that’s your life”.

Participant L: “I was there, at home, I suffered, I resisted, sticking together for the good of the family, but inside… I was wrong because now my family is happier, even if -technically- we are not a family.”

Participant F: “Well, [it is difficult] to realise what is happening, not to put off … you keep saying yourself that something will change, that in any case, everything will be fine, or that it’s your fault. No, absolutely! You need to ask help, help, help, until something happens. However, I know that it’s not easy when there are children, when there is no money, but once you start your trip, then things happen.”

Personal values or negative emotions could hinder the women's awareness of being a victim of IPV. For example, the idea of “family cohesion” (i.e., the belief that children need not-separated parents or that divorce is a big personal failure) was experienced as a strong barrier for some women, exactly like the belief that that family situation may eventually change.

Participant I: “However, it’s always a piece of life that you share with another person with whom you hoped to grow old. If it doesn’t happen, it’s a failure, because I see the divorce as a failure.”

Participant C: “I stayed, hoping that he would change.”

As to negative emotions, fear was a strong barrier: Women were afraid of not being able to take care of children on their own without a job and a house to live in; but they were also afraid of the partner’s reaction, which could worsen the situation and endanger their own and their children’s lives.

Participant E: “You have fear: What can I do with the children? What can I do? Where can I go? How can I support them?”

Participant D: “Because I was sure to protect her [daughter]: I kept him quiet to protect her.”

However, reaching the point of no return or a specific event, like an extremely violent episode or a request from the children, brought the final awareness to victims of IPV that something had to be changed (*knowledge*).

Participant I: “It is different, talking about what happened two years ago and talking about the terror that a woman feels when she enters in her house, and she knows that at the slightest misstep, he can hit you at any moment. So, this keeps you from doing anything, because you have to live in that house. Then, when you start realising—I don’t know where I found that courage that day-you take the children and go away because you have reached the limit.”

Participant C: “Yes, he was violent at home and my oldest daughter, who was five at that moment, asked me to send her father away because she couldn’t stand it anymore.”

Women reported that experiencing IPV not only affected their *knowledge*, but also reduced their *self-efficacy* and their ability to gain access to *community resources*. There was a family dynamic that confined women to the home and in a subordinate position, while men were part of society with their position as bread-winners, rulers of their house and family. For this reason, men prevented their partners from going to work or managing family affairs (e.g., documents, children's medical visits, etc.). Use of verbal abuse also contributed to lowering the women’s self-esteem, self-efficacy and reduce them to social isolation. The women usually blamed themselves for their “subordinate” position, but through the caring relationship established with the professionals at the SCW, they gradually realised that their “oppressed” position was caused by their violent partner, and did not depend on their own personality or skills (*self-efficacy*). As such, the caring relationship interrupted a self-blaming process that prevented them from seeing any alternatives.

Participant E: “This person leads you to think that you cannot manage, that you’ll never manage, that you are nothing, that you are not worth anything, that you are not capable of, etc. etc.”

Consistently, women understood that forced isolation also reduced social support (*community resource*) as well as access to information, thus reducing the opportunities for developing new abilities (*knowledge and skills*). Once women realised this, they asked for support to improve their professional competences and become economically independent (*skills*).

However, even when the “recovery” process worked well, and self-esteem was repaired, women still felt insecure and vulnerable in some spheres of life, reporting difficulties with trust and intimate relationships.

Participant F: “To be honest, I still have some difficulties as a woman! I still have to work on relationships. Once I met a very nice guy on the Internet, but I ruined everything because I’m still full of fears, I do not know how to behave.”

*Which obstacles do survivors commonly face in growing or accessing resources in each of these areas?* Women victims of IPV encountered numerous difficulties. The first difficulty was establishing trust. Women quite often felt judged or not believed by people who should have helped them, thus making it harder to turn to dedicated services to obtain information (*knowledge*) as well as social support. When they decided to finally access the services, they were still doubtful.

Participant I: “However, when I arrived here, at first I was concerned because I asked myself “Will they help me? Won’t they?” Because I had heard that they helped several women, but also that they maybe weren’t able to help others.”

Participant C: “Despite the fact that you explain [your situation], it seems that you come looking for something, I don’t know, to cheat people.”

Emotions like shame, fear, and anger emerged from the interviews. Emotions could hamper the process of “recovery” and women could be easily “overwhelmed” by negative emotions.

Participant A: “I was afraid of not seeing my children any more, that he’d take them away, but they [the SCW] guaranteed to me that nobody would take my children, that they would be with me, and that nobody would hurt them.”

Participant F: “That is, I always felt this, this anguish, this burden that I would still need him because I can’t manage on my own.”

Given this emotional burden, women recognised that the first approach was critical and that special care from, as well special qualities in the person who establishes the first contact (and who is in charge of continuous support), are needed.

Participant B: “Yes, maybe to be more sympathetic with people who have difficulties in expressing, in escaping from their situation. I had to be good at, all the bits of help could be good, but you also have to find a good psychologist."

Participant G: “They could be more sympathetic, a little bit kinder, the first meeting should have been a little bit kinder for me.”

Several women admitted that they had problems with the *solutions* offered by the SCW: For example, moving to a secure house and living with other women with whom the only thing they had in common was being a victim of violence. Co-housing was difficult, both for cultural and structural reasons (e.g., cultural diversity, lack of private space, forced intimacy).

Participant G: “Yes, it was positive, but you need self-control “Yes, it was positive, but you also need self-control—if there were only us women insults and arguments could happen, but when there are children involved you need to be more careful and avoid having words with someone, and it can become difficult even to say “your child has done this to mine” for example, "your son has done this to mine”. It’s difficult.”

Participant A: “There was a window, and we spent all day by the window. It was only one room. In that room there was a bed, a kitchen, and a big door…”

Only a few women recognised that co-housing could also be enriching:

Participant B: “Well, I think that everyone wishes to stay with their family if the family is as it should be. In that situation, it [the safe house] was useful because we could talk with other women, and we enriched each other with other cultures.”

Most of the women doubted that safe houses were an ideal solution for children, and those who were able to—usually those with stronger support networks and easier access to community resources—looked for and found other solutions.

Participant E: “Yes, they offered me a safe house, but I rejected it because I preferred to go to my parents in [another region] to try to traumatise my children as little as possible. As there was this possibility, we worked it out.”

There were also many contextual obstacles that reduced the women’s capacity to grow and pursue their goals: bureaucracy, unemployment, structural barriers to women employment could make the efforts of the women and of the SCW useless, and the experience of the women very frustrating. Investing many hours in an internship without the security of obtaining a job, or having no guarantee of having a house or a job for a long time could be a disempowering experience.

Participant B: “At the end of the course, it was not guaranteed to have a job. However, some of us obtained a little job, but not many.”

Participant G: “I would at least like go to bed without thinking that at any moment, I could end up on the street.”

### Impact

The capacity of the program for women victims of IPV to produce empowerment involves reflecting on one’s progress, both in terms of internal experience and external change, and on other unexpected (positive or negative) consequences. The accounts of women during the interviews clearly show that they had the chance to reflect on their own story, understand the psychological and contextual barriers that they faced, that sometimes stopped them, but in other cases they were able to overcome. Participants demonstrated a deep *knowledge and understanding* of their history of violence, the role that it had, and its effects in their current situation.

Two women summarised their process of empowerment as a second life.

Participant I: “It is like I was “reborn”.

There was a life in which they were victims of IPV, and for which they blamed themselves and felt disappointed and worthless. Then, there was a second life, in which they were women with difficulties and past-related problems, but with the awareness that they could solve their problems. They developed a new perspective in which taking care of their children’ health, being a single mother, obtaining a job, and falling in love (again) was possible.

Victims of IPV acknowledged the role of the SCW as being able to provide a compassionate and non-judgmental environment, representing both a landmark and a milestone in the women’s experience, thus contributing to their process of change. Moreover, some of the women recognised that they have the power to control their lives, and that that the support they received helped them to recognise it.

Participant L: “So, I have learnt a lot, I have become stronger, I don’t know…yes, it is a total change… they helped me, I would never deny that… but… they don’t need to follow you around all the time, you become stronger yourself…”

For some women telling their history of IPV was hard, because in doing so they had the opportunity to realise what they went through:

Participant E: “I don’t want to remember the bad moments of my life […] I wasted time, energy and everything. For me it's not good, I do not feel well now talking about these things, it’s not easy what I’ve been through.”

In this case, negative feelings prevented the woman from seeing a clear future and from maintaining continuity between those bad moments and the present/future.

Women reflecting on their own experience identified some important outcomes that they were able to achieve: stopping (enduring) violence, protecting their children, offering them a “normal” environment, re-establishing a network of relationships, gaining self-esteem and psychological and economic autonomy.

In most cases they arrived at the SCW asking for protection, emotional, and material support, and they found some answers. The offer of psychological and emotional support from SCW was important for women victims of IPV, when other needs were prioritised the role of the other services was deemed to be critical.

#### Beyond system-defined outcomes, how do various services affect outcomes most important to survivors?

The collaboration between different services was useful to guarantee protection, to distribute resources, and to direct women victims of IPV to the services that were appropriate to meet their personal requests (e.g., tutoring for children and Child Protection Services, economic help, safe houses, legal consultancy, general information) and employment needs (e.g., internships for professional positions). But this process was not simple, and not equally effective for all the women. The women appear well aware of this, with a clear understanding of the importance of contextual resources and opportunities in the empowerment process. Economic independence, job qualifications, an occupation and protection were identified as important outcomes for the women. However, their meaning and their relevance may change as long as their “recovery” process is taking place. And despite the efforts of SCW, the outcomes that were relevant from the women’s perspective were not always recognised as equally “relevant” by other services/organisations. Social services and law enforcement were blamed for their judgmental attitudes. Two women felt judged and blamed by social workers, and several participants felt that the effort of the Police authorities to give them protection was strong at the beginning, but less significant over time.

Participant D: “Initially after the restraining order, I felt protected… But afterwards I didn't feel safe anymore because I saw that he could come home when he wanted, and he did.”

In all those cases women were still happy with the SCW, but described it as a "weak" organisation, underfunded and that has to rely (too much) on the collaboration of other services, which are not equally “engaged” in helping IPV victims to advance their empowerment process.

### Discussion

The aim of the present study was to evaluate the empowerment capacity of the program for IPV victims offered by a SCW located in the Centre-North of Italy. The request was rooted in the willingness of the SCW to improve its service, to identify its strengths and weaknesses, and to understand its contribution to women’s empowerment. To address these goals, we adopted the EPM model—a robust theory-based general framework for the design and evaluation of programs addressing IPV [8, 9]—to analyse the narrative accounts of ten women survivors of IPV reporting their experience with the service provided by a SCW.

Participants were young women and mothers. The majority of them had experienced several years of physical and psychological violence before asking for help from the SCW. This usually happened after an extremely violent episode, which the women themselves often defined as the turning point which made them aware of their life condition. Seeking help is a complex process for victims of IPV: Findings from this study confirm that economic and psychological dependence on the abusive partner are among the obstacles that contribute most to slowing down the process of seeking help, in line with what was found by Anderson and Saunders [2].

#### The empowerment process

According to women’s accounts, the SCW did not have a predefined program, but goal formation was personalised and changed over time in relation to the women’s needs and desires. This seems coherent with the need to incorporate self-determination into empowerment programs, as claimed by Kasturirangan [18].

The majority of women reported that at first they felt alone, disorientated and compelled to end violence (e.g., by moving away from the violent perpetrators). Stopping violence was a *sine qua non* condition for any other achievement: Self-actualisation came later and could have a different focus (e.g., obtaining a job, becoming economically independent, and living in a safe house with children). Thus, the SCW first offered psychological support, safe shelter and protection (in cooperation with other services). The capacity of the SCW to offer emotional support in a non-judgmental way was recognised as its main strength. This finding is coherent with other evidence collected in advocacy programs for women victim of abusive partners [1].

Subsequently some women realised that they could also benefit from internships, legal consultancy, economic help, and social support. The achievement of these goals depends on individual and social resources, as well as on psychological strengths and on structural barriers, and relies on the capacity of the system to help women put together their own puzzle, offering or building the pieces that they need [18]. Indeed, some of those pieces are part of the psychological empowerment process (e.g., gaining knowledge, control, and self-esteem), but others require different structural conditions (e.g., supporting women’s access to the job market, reducing social stigma for victims of IPV etc.), involving wider societal change.

The capacity of different agencies to work together and to establish good operational partnerships is critical to provide women with a context that allows them to pursue their own goals. When women saw that the different nodes of the institutional networks worked well and established a non-judgmental environment, they felt more secure and protected, and this “baseline” condition helped them to go on and restart their lives. When their experience was somehow contradictory, with different institutions paying attention to the women's experiences and needs in inconsistent ways, it was more difficult for women to attain their goals, thus reducing the impact of the empowerment process [9]. This finding is consistent with previous studies that show that the systems’ response to victims of IPV can reinforce the experience of violence, in particular when they are inadequate (e.g., lack of recognition, insufficient protection) [4, 21]. To use a metaphor, we could say that a good operational partnership between institutions is like a cement that keeps the stones of a paved path close together. If there are holes between the stones it is not impossible to follow the path, but the risks of tripping and having to stand up again are much more concrete. Economic resources is another important factor that keeps the stones of the path close together. However, SCWs in many countries, including Italy, receive limited financial support from the State or local government, thus weakening the power of those organisations [7].

Most women experiencing violence were continually belittled, which reduced their self-esteem and self-efficacy. Some reported being isolated, and/or voluntarily retreating from social relationships and the community, which limited their opportunities to access community resources and created serious difficulties for them in asking for help. This picture is coherent with those provided by other scholars [4]. The interviews clearly showed that women, with the help of the SCW, could become increasingly aware of the forms and the mechanisms of violence, and as a result stop blaming themselves for their situation. These results go along with those obtained in various countries showing that women accessing Support Centres report an increase in their self-esteem, self-awareness, empowerment, and well-being [27].

The support service helped women with legal issues, and facilitated access to the opportunities offered by other social and community services. Provision of information appeared as central in the process of empowerment and in the SCW programs. This result resonates well with those of Allen et al. [1] and those of Sullivan et al. [37] who found that women, by participating in the activities proposed by the SCWs, could also develop significant relationships with other women victims of IPV and acquire concrete skills that contribute to the development of self-esteem [36].

The impact of the SCW program on the victims’ lives was positive, as participants themselves defined the Centre as a landmark in their lives. It was recognised as important due to being a welcoming place as well as for offering useful psychological support, regardless of the fact that it could not on its own provide the economic resources which the women needed, such as workplaces or bigger houses. This is a problematic issue that is common for SCWs in Italy [7]. Nevertheless, the SCW represented for some of the women a point of access to a network of institutions and services that could provide those resources. When the network worked well, offering resources and providing a non-judgemental environment, the SCW was recognised as a booster for women’s empowerment, but in many circumstances this process was neither straightforward nor complete, thus reducing the opportunities for women to reach the desired outcomes.

#### Strengths, limitations, and implications

One of the strengths of this study is to evaluate the activity of a SCW in Italy by using a theory-based framework that is coherent with the empowerment principles that underlie its activity [8, 9]. By adopting a formative evaluation approach, we have focused on how SCW interventions are experienced by participants, relying on the perspective of women survivors of IPV to understand the resources and limits of the SCW and its collaborations with other service providers. This work showed that evaluation studies can benefit from qualitative approaches that produce rich data and shed light on users’ experience, and can simultaneously allow users to examine how the services impacted on their story, as well as identifying positive and negative aspects/elements of their experiences. In this sense conducting research with and within SCW may represent an additional opportunity for women who have escaped IPV to deepen the reflective process that they started with the SCW, constructing and sharing a different narrative. The importance of this process should not be underestimated: according to Rappaport [29] empowerment could also be defined as a process where tales of terror are turned into tales of joy.

To our knowledge, there is not much literature that has evaluated the activity of Support or Anti-Violence centres in Italy using an empowerment theory-based framework. Moreover, most of the literature on the evaluation of the activity of support services for IPV victims measured the outcomes quantitatively, which may fail to adequately capture the subjective perspective of service users on how programs are delivered. Moreover, by explicitly recognising the capacity of women survivors of IPV to know what they need and to evaluate the quality of the answers they receive, an empowerment theory-based approach to evaluation can contribute to women’s empowerment, thus becoming a structural part of the support program. Finally, this is one of the few evaluation studies of programs targeting IPV victims that is explicitly theory-driven, thus offering an empirical test of the validity and applicability of the empowerment process model in the Italian context.

Despite these strengths, some of the limits of this work should also be acknowledged. The analysis presented here is based on a small sample and included participants who were at different points of their story of violence (i.e., a distant past, a recent past, a current situation in which violence has been interrupted but they are still engaged in ongoing legal disputes with ex-partners). The limited number of interviews and the specificity of women’s story at the moment of data collection could limit the generalizability of the results. Nonetheless these data offer important information, and the diversity of the stories favours a better understanding of IPV program implementation. A further limit is due to the fact that the sample was to some extent self-selected. The women who accepted to be interviewed were still in contact with the professionals of the SCW, even those who “completed” their program. Thus, we collected information only from women who had constructed and maintained an overall positive relationship with the SCW, whereas we were not able to reach those women who did not find any support at the Centre or did not trust its work. Even though we are aware of the risks of a positive selection bias, the women’s accounts did however show that they were not reluctant to address the limits both inside and outside the activity of the SCW. To the contrary, women’s accounts revealed a highly critical understanding of their experience within the institutional contexts, as well as their ability to identify several limits of the interventions of different institutions. Although other informants with different experiences of the service could provide different accounts, potentially useful for capturing other details for improving social interventions and service on IPV, participants in this study provided a consistent picture of the support they received, in terms of both strengths and weaknesses.

Finally, results of the present study highlighted several implications for practice. First, the SCW has as its main strength offering emotional support and information to women victims of violence. This seemed a key element, and a starting point for increasing women’s self-esteem, self-efficacy, awareness of their situation, and community resources. The program offered women the opportunity to engage in a process that allowed them to gain more control over their lives, leading them to count on themselves and on their own abilities to mobilise contextual resources and to activate, in terms of the EPM model [9], an empowerment process. Indeed, even when women were frustrated because they did not obtain what they expected (e.g., a qualified job, an affordable house that meets their family needs), they recognised the progress that they made in their lives and the key role of the SCW. This shows that it is necessary to promote and to strengthen the activities of the SCW, providing adequate resources. Second, it is important to act on a socio-political level by facilitating access to qualifications, training, and job opportunities for IPV victims, but also by offering specialized training to other professionals who work with victims of IPV and who may display a judgmental approach. Third, it may be important to increase the quality of collaboration between social and health services. When women feel that different institutions involved in IPV intervention effectively collaborate they feel more accepted and protected. Moreover, effective partnership between services increases the capacity of services to meet women’s needs. Supporting the capacity of the institutions to work together and to establish partnerships with common training activities, facilitation of collaboration, and reciprocal trust, is pivotal to support women’s empowerment [10]. Fourth, as IPV leads to social isolation for women, it is important for services to increase their visibility and become more accessible to the community (e.g., through social media, or primary care physicians). Lastly, as women victims of IPV are often also mothers, prevention programs and intervention should take into account children’s conditions and needs, that are deemed as critical by the women themselves in evaluating the quality of the support provided.

## Conclusion

In conclusion, by adopting the EPM model [9] as a reference framework, this study shows that the program offered by SCW to women victims of IPV favours the empowerment process of women through the acquisition of knowledge, skills, self-efficacy, and by improving their integration in the social context in term of protection, security, and health. Structural limitations related to the system’s difficulties in ensuring equal access to the service for all women, and in providing them with legal and material support, are recognised by women victims of IPV as major barriers to their empowerment process. Future interventions in this direction are recommended.

## Supplementary Information

**Additional file 1**. Interview guidelines.

## Data Availability

The datasets generated and analysed during the current study are not publicly available due participants’ privacy concerns but are available from the corresponding author on reasonable request.

## Abbreviations

IPV: Intimate Partner Violence
EPM: Empowerment Process Model
SCW: Support Centre for Women

## References

[1] Allen NE, Larsen S, Trotter J, Sullivan CM (2013). Exploring the core service delivery processes of an evidence-based community advocacy program for women with abusive partners. J Community Psychol.

[2] Anderson DK, Saunders DG (2003). Leaving an abusive partner: an empirical review of predictors, the process of leaving, and psychological well-being. Trauma Violence Abuse.

[3] Associazione Italiana Psicologia. Codice etico. 2015, March, 27. https://aipass.org/en/node/11560.

[4] Bostock JAN, Plumpton M, Pratt R (2009). Domestic violence against women: understanding social processes and women's experiences. J Community Appl Soc Psychol.

[5] Brodsky AE, Cattaneo LB (2013). A transconceptual model of empowerment and resilience: divergence, convergence and interactions in kindred community concepts. Am J Community Psychol.

[6] Buckner JC, Beardslee WR, Bassuk EL (2004). Exposure to violence and low-income children's mental health: direct, moderated, and mediated relations. Am J Orthopsych.

[7] Carrano T, Romito P, Folla N, Melato M (2017). I centri anti-violenza [Anti-violence centres]. Violenze su donne e minori: una guida per chi lavora sul campo. Nuova edizione [Violence against women and children: a guide for those working in the field. New version].

[8] Cattaneo LB, Chapman AR (2010). The process of empowerment: a model for use in research and practice. Am Psychol.

[9] Cattaneo LB, Goodman LA (2015). What is empowerment anyway? A model for domestic violence practice, research, and evaluation. Psychol Violence.

[10] Cicognani E, Albanesi C, Valletta L, Prati G (2020). Quality of collaboration within health promotion partnerships: Impact on sense of community, empowerment, and perceived projects' outcomes. J Community Psychol.

[11] Cody MW, Jones JM, Woodward MJ, Simmons CA, Gayle Beck J (2017). Correspondence between self-report measures and clinician assessments of psychopathology in female intimate partner violence survivors. J Interpers Violence.

[12] European Union. Agency for Fundamental Rights, Europäische Union Agentur für Grundrechte, & FRA-European Union Agency for Fundamental Rights. Violence against women: An EU-wide survey: Main results. FRA, European Union Agency for Fundamental Rights. 2014.

[13] Evans SE, Davies C, DiLillo D (2008). Exposure to domestic violence: A meta-analysis of child and adolescent outcomes. Aggress Violent Behav.

[14] Franchek-Roa KM, Tiwari A, Lewis-O’Connor A, Campbell J (2017). Impact of childhood exposure to intimate partner violence and other adversities. J Korean Acad Child Adolesc Psych.

[15] García-Moreno C, Jansen HAFM, Ellsberg M, Heise L, Watts C (2005). WHO multi-country study on women’s health and domestic violence against women,.

[16] ISTAT. Annual report 2014—the state of the Nation. Primaprint srl. Viterbo. 2014.

[17] Israel BA, Schulz AJ, Parker EA, Becker AB (1998). Review of community-based research: assessing partnership approaches to improve public health. Annu Rev Public Health.

[18] Kasturirangan A (2008). Empowerment and programs designed to address domestic violence. Violence Against Women.

[19] Kokka A, Mikelatou M, Fouka G, Varvogli L, Chrousos GP, Darviri C (2019). Stress management and health promotion in a sample of women with intimate partner violence: a randomized controlled trial. J Interpers Violence.

[20] Larkin M, Watts S, Clifton E (2006). Giving voice and making sense in interpretative phenomenological analysis. Qual Res Psychol.

[21] Lelaurain S, Graziani P, Lo Monaco G (2017). Intimate partner violence and help-seeking: a systematic review and social psychological tracks for future research. Eur Psychol.

[22] Lewis T, Kotch J, Thompson R, Litrownik AJ, English DJ, Proctor LJ, Dubowitz H (2010). Witnessed violence and youth behavior problems: a multi-informant study. Am J Orthopsych.

[23] McDonald R, Jouriles EN, Ramisetty-Mikler S (2006). Estimating the number of American children living in partner-violent families. J Fam Psychol.

[24] Orang T, Ayoughi S, Moran JK, Ghaffari H, Mostafavi S, Rasoulian M, Elbert T (2018). The efficacy of narrative exposure therapy in a sample of Iranian women exposed to ongoing intimate partner violence—A randomized controlled trial. Clin Psychol Psychother.

[25] Patton MQ (1997). Utilization-focused evaluation.

[26] Pels T, van Rooij FB, Distelbrink M (2015). The impact of intimate partner violence (IPV) on parenting by mothers within an ethnically diverse population in the Netherlands. J Family Violence.

[27] Pomicino L, Beltramini L, Romito P (2019). Freeing oneself from intimate partner violence: a follow-up of women who contacted an anti-violence center in Italy. Violence Against Women.

[28] Rappaport J (1987). Terms of empowerment/exemplars of prevention: Toward a theory for community psychology. Am J Community Psychol.

[29] Rappaport J (2000). Community narratives: tales of terror and joy. Am J Community Psychol.

[30] Saunders RP, Evans MH, Joshi P (2005). Developing a process-evaluation plan for assessing health promotion program implementation: a how-to guide. Health Promot Pract.

[31] Schauer M, Schauer M, Neuner F, Elbert T (2011). Narrative exposure therapy: a short-term treatment for traumatic stress disorders.

[32] Simmons SB, Knight KE, Menard S (2018). Long-term consequences of intimate partner abuse on physical health, emotional well-being, and problem behaviors. J Interpers Violence.

[33] Smith JA, Shinebourne P (2012). Interpretative phenomenological analysis.

[34] Sprague S, Scott T, Garibaldi A, Bzovsky S, Slobogean GP, McKay P, Swaminathan A (2017). A scoping review of intimate partner violence assistance programmes within health care settings. Eur J Psychotraumatol.

[35] Stewart DE, Vigod S, Riazantseva E (2016). New developments in intimate partner violence and management of its mental health sequelae. Curr Psychiatry Rep.

[36] Sullivan CM (2011). Evaluating domestic violence support service programs: waste of time, necessary evil, or opportunity for growth?. Aggress Violent Beh.

[37] Sullivan CM, Baptista I, O’Halloran S, Okroj L, Morton S, Stewart C (2008). Evaluating the effectiveness of women’s refuges: a multi-country approach to model development. Int J Comp Appl Crim Just.

[38] Trabold N, McMahon J, Alsobrooks S, Whitney S, Mittal M (2020). A systematic review of intimate partner violence interventions: state of the field and implications for practitioners. Trauma Violence Abuse.

[39] Trabold N, O'Malley A, Rizzo L, Russell E (2018). A gateway to healing: a community-based brief intervention for victims of violence. J Community Psychol.

[40] Zimmerman MA (1995). Psychological empowerment: Issues and illustrations. Am J Community Psychol.

