# Supplementary material for: Evaluating interventions with victims of intimate partner violence: a community psychology approach
Source: BMC Womens Health. 2021 Apr 6;21:138. doi: 10.1186/s12905-021-01268-7 (PMC8025317; doi:10.1186/s12905-021-01268-7)
Supplement: Supplementary file 1 — Additional file 1. Interview guidelines. [file 12905_2021_1268_MOESM1_ESM.docx]

Interview Grid

Buongiorno siamo del dipartimento di Psicologia e stiamo svolgendo un progetto di ricerca in collaborazione con il Centro Donna di Forlì con l’obiettivo di comprendere meglio punti di forza e di debolezza del servizio offerto dal centro per quanto riguarda i percorsi di protezione. A noi questo servirà anche per la nostra tesi di laurea magistrale. I dati che ricaveremo da queste interviste saranno trattati in forma anonima e col solo scopo di comprensione. Le faremo alcune domande relative al percorso di protezione che lei ha affrontato e le chiediamo di poter registrare questa intervista per mantenere traccia ed evitare di perdere alcune informazioni rilevanti.

Good morning we are from the Psychology department, of the University and we are carrying out a research project in collaboration with the Women's Center of Forlì with the aim of better understanding the strengths and weaknesses of the service offered by the center with regard to protection paths. I’m XX and will conduct the interview. The data collected will also be used for the master's thesis of XX, who is with me today and will help with the analysis . The data we will obtain from these interviews will be treated anonymously and with the sole purpose of understanding. We will ask you some questions related to the protection path you have gone through and ask your permission to audio record this interview to keep track and avoid losing any relevant information.

DEMOGRAPHICS

1. Quanti anni ha?
2. Da dove viene?
3. Dove vive ora?
4. Da dove viene la sua famiglia di origine?
5. Dove vive ora la sua famiglia?
6. Lavora? Se si che lavoro fa?
7. Lavorava anche prima e durante il percorso di protezione?
8. Che scuola ha fatto?
9. How old are you?
10. Where does it come from?
11. Where do you live now?
12. Where does your family of origin come from?
13. Where does your family live now?
14. Do you work? If so, what do you do?
15. Did you also work before and during the protection process?
16. What school did you go to?

1° AREA:

*Inizio del percorso di protezione*

1. Quando è avvenuto il primo contatto con il Centro Donna?
2. Prima del primo contatto aveva mai sentito parlare del Centro Donna?
3. C’è stato qualcuno che l’ha indirizzata in questo centro?
4. Con quali operatrici ha parlato al Centro Donna?
5. Per quale motivo si è rivolta al Centro Donna?
6. Prima del percorso che ha concluso aveva già avuto contatti con questo centro che però non ha portato avanti?

- Perché?
- L’ha voluto interrompere lei?

1. Le avevano proposto altri servizi diversi dal Centro Donna?
2. All’inizio di questo percorso di protezione è entrata direttamente in contatto col Centro Donna o c’è stata una collaborazione con più servizi?

- Con quali operatori è entrata in contatto?
- Quale tipo di aiuto ha ricevuto?
- Come le sono sembrati gli operatori nei suoi confronti? (chiedere un episodio)
- Ha avuto l’impressione di potersi fidare?
- Si è sentita sostenuta/protetta?
- Si è sentita compresa?
- È riuscita da subito a raccontare agli operatori la sua storia?
- Come le è sembrato il lavoro di collaborazione tra i servizi?

1. Com’è arrivata al percorso di protezione?
2. Ci potrebbe raccontare come si è svolto questo percorso di protezione?

- È andata come si aspettava?
- Son stati soddisfatti i bisogni che sentiva di avere?
- Si aspettava una risposta diversa?

1. Il percorso che ha svolto le era stato delineato da subito in maniera chiara?
2. C’è stato qualcosa per cui il percorso prospettato in partenza è cambiato?

- Perché?
- Cosa è cambiato?
- Come si è sentita a causa di questi cambiamenti?

1. Ha avuto momenti in cui ha pensato di non farcela durante il percorso? (quali e perché)
2. In contemporanea con il servizio che le è stato offerto dal centro, ha avuto sostegno anche da altri? (chi?)
3. Durante il percorso ha avuto contatti con altre donne nella sua stessa situazione?

- È stato positivo o negativo? Perché?

Beginning of the protection path

1. When did the first contact with the Women's Center take place?

2. Had you ever heard of the Women's Center before the first contact?

3. Did someone referred you to this center?

4. With which operators did you speak at the Women's Center?

5. Why did you contact the Women's Center?

6. Did you already have any interrupted contact with this center in the past?

- Why? Was it you decision?

7. Did they offer you other services other than the Women's Center?

8. At the beginning of this protection path did you come into direct contact with the Women's Center or was there a collaboration with several services?

- With which professionals did you come into contact?
- - What kind of help did you get?
- - How did the professionals behave towards you? (ask for an episode)
- - Did you get the impression you could trust them?
- - Did you feel supported / protected?
- - Did you feel understood?
- - Were you able to tell the professionals your story straight away?
- - How did the collaboration between services seem to you?

9. How did you arrive at the protection path?

10. Could you tell us how this protection process took place?

- Did it go as you expected?

- Were the needs you felt met?

- Did you expect a different answer?

11. Was the path you followed immediately outlined in a clear way?

12. Was there something that changed the route planned at the start?

- Why?

- What has changed?

- How did you feel about these changes?

13. Did you have moments when you thought you wouldn't make it along the way? (which and why)

14. At the same time as the service offered to you by the center, did you also receive support from others? (who?)

15. Did you have contact with other women in the same situation as you did?

- Was it positive or negative? Because?

*Uscita dal percorso*

1. Quant’è durato il percorso?
2. Come si è concluso il percorso?
3. Cosa ha permesso la conclusione?
4. Come ha vissuto questa esperienza?
5. Che tipo di contatto è rimasto con l’organizzazione una volta concluso il percorso?

Ending the protection path

1. How long did the protection path last?
2. How did it end?
3. What allowed the conclusion?
4. How did you live this experience?
5. What kind of contact remained with the organization once the path was completed?

2°AREA: Figli minorenni

1. Può raccontarci qualcosa dei suoi figli?

- Quanti figli ha?
- Quanti anni ha/hanno?
- Come si chiama/chiamano?

1. Quanti anni aveva suo figlio quanto siete stati inseriti nel percorso di protezione?
2. Suo figlio ha mai assistito agli episodi di violenza?

- Come pensa che suo figlio abbia vissuto la situazione?

1. Suo figlio conosce i motivi per cui è stato iniziato questo percorso?

- Come ha provato a spiegarglielo?

1. Suo figlio è rimasto con lei durante tutto il percorso?
2. Come va a scuola? (compagni/insegnanti/voti)

- Ci son stati problemi scolastici e/o di comportamento prima o dopo il percorso?

1. Come crede che si sia sentito suo figlio durante il percorso?
2. Secondo lei, che conseguenze ha avuto il percorso su suo figlio?
3. Ha notato dei cambiamenti in suo figlio?

- Se sì, c’è stato un momento in particolare in cui ha visto che il bambino è cambiato?
- Se sì, cos’è successo?

1. In che modo la presenza di suo figlio ha orientato la sua scelta di entrare nel percorso di protezione?
2. Se non avesse avuto figli avrebbe preso decisioni diverse?
3. Pensa che i servizi abbiano soddisfatto anche le necessità di suo figlio?
4. Aver dovuto prendere queste scelte come la fa sentire in qualità di donna e di madre?
5. Cosa fanno e come stanno adesso i bambini?

2 AREA Children under legal age

1. Can you tell us something about your children?

- How many children do you have?

- How old are they?

- What are their names?

2. How old were your children when were you included in the protection process?

3. Have your children ever witnessed the violence?

- How do you think your children experienced the situation?

4. Do your children know the reasons why this journey was started?

- How did you try to explain it to them?

5. Did your children stay with you throughout the journey?

6. How is school going? (classmates / teachers / grades)

- Were there any school and / or behavior problems before or after the protection path?

7. How do you think your son felt along the way?

8. In your opinion, what consequences did the protection path have on your children?

9. Have you noticed any changes in your children?

- If so, was there a particular moment in which you saw those changes?

- If so, what happened?

10. Did the presence of your children influenced your decision to enter the protection path?

11. If you hadn't had children, would you have made different decisions?

12. Do you think the services also met your children’s needs?

13. How does having to make these choices make you feel as a woman and as a mother?

14. What are the children doing and how are they now?

3°AREA: Partner

1. Si è sentita protetta dalla minaccia del partner una volta iniziato il percorso?
2. Si è sentita protetta durante il percorso ed alla sua conclusione?
3. Ha riavuto contatti col suo partner?

- Per quali motivi?

1. Che lei sappia, il suo partner ha svolto qualche percorso di sostegno?

3° Area: Partner

1. Did you feel protected from the threat of your partner once you started the protection path?

2. Did you feel protected during the process and at its conclusion?

3. Did you keep in touch with your partner?

- For what reasons?

4. To your knowledge, has your partner been on any support path?

Chiusura:

Ci son state situazioni in cui lei si è sentita utile per altre donne?

Può raccontarci un episodio in cui ha capito di avercela fatta?

Alla luce della sua esperienza, se un domani una donna dovesse trovarsi nella stessa situazione che consigli le darebbe? Come le aiuterebbe?

Closing questions.

Were there any situations in which you felt useful for other women?

Can you tell us an episode in which you realized you “made it”?

In light of your experience, what advice would you give to a woman in your same situation? How would that help her?
